# Statistical Optimization and Analysis of Factors Maximizing Milk Productivity

**DOI:** 10.3390/ani15101475

**Published:** 2025-05-20

**Authors:** Yücel Kurtuluş, Hasan Şahin, Abdulkadir Atalan

**Affiliations:** 1 Department of Industrial Engineering, Graduate School of Education, Bursa Technical University, Bursa 16310, Türkiye; yucel.kurtulus@hotmail.com; 2 Department of Industrial Engineering, Faculty of Engineering and Natural Sciences, Bursa Technical University, Bursa 16310, Türkiye; h.sahin@btu.edu.tr; 3 Department of Industrial Engineering, Faculty of Engineering, Çanakkale Onsekiz Mart University, Çanakkale 17020, Türkiye

**Keywords:** milk yield, dry matter, regression analysis, variables, optimization

## Abstract

This study was conducted to determine the biological and environmental factors affecting milk yield and dry matter consumption and to analyze the effects of these factors on animal production. The study determined the variables affecting milk yield as input factors, such as lactation period, number of days of gestation, age, TMR dry matter ratio, and environmental factors. As a result of regression analyses, it was determined that each 1% increase in the TMR dry matter ratio decreased the milk yield by 0.9148 L, and each increase in the number of lactations increased the daily milk yield by 3.753 L. However, it was observed that the increase in the number of lactation days caused a decrease in milk production, and milk yield decreased as the gestation period extended. The most appropriate independent variable values were determined using statistical optimization analyses to maximize milk yield and optimize dry matter consumption. As a result of the analyses, the optimum value for the TMR dry matter ratio was calculated as 46.77%, 5 for lactation number, 6 for lactation day number, 230 days for gestation period, 55.8 months for cow age, and 20 °C for air temperature. The optimum values of the dependent variables were determined to be 61.145 L for daily milk yield and 19.033 units for dry matter consumption. The prediction intervals provided by the model served as reference points for future observations and showed that milk production was strongly affected by certain environmental and biological factors.

## Simple Summary

This study investigated the effects of biological, environmental, and nutritional factors on increasing milk yield and making feed use more efficient in dairy cows. The study examined the role of animal age, birth period, air temperature, feed rate, and lactation period on milk production. Data were collected from a large-scale farm in Turkey and analyzed using statistical optimization methods. According to the results, milk production is significantly affected by the animal’s physical characteristics and external conditions such as air temperature. Given this information, the model developed determined the ideal conditions to maximize milk production while minimizing feed consumption. This study provides concrete and applicable recommendations that will help farmers increase productivity, reduce costs, and protect animal welfare. The results also have significant social importance regarding sustainable animal husbandry and food security.

## 1. Introduction

Milk production is strategically important today to meet the increasing global food demand [1]. Especially in developing countries, increasing population and changing dietary habits increase the demand for animal foods and necessitate the development of sustainable production methods [2]. Improving milk production efficiency is critical to economic sustainability and food security [3]. Various studies have been conducted on genetic improvement, feeding strategies, and management techniques to increase productivity, and most of the studies consider the factors independently and use multivariate optimization methods [4,5].

Generally, there are three main cattle breeds regarding milk yield: native, cultured crossbreed, and cultured [6]. Occasionally, large-scale individual and government-supported farms have been established, where thousands of milking cattle are kept [7]. Studies on selection and environmental temperature conditions that have lasted for years have increased dairy cattle productivity [8,9]. Still, despite all these studies, the same increase in milk yield has not been observed [10,11]. Milk productivity is critical to increasing and optimizing farm performance in modern cattle farming [12]. With the modernization of agriculture and animal husbandry, data analytics and statistical methods in production processes are becoming widespread [13]. Statistical analyses play an important role in determining the factors affecting milk productivity and integrating these factors into management strategies [14]. Statistical methods allow us to understand the relationships between multiple variables using detailed analyses performed on large datasets [15].

Linear regression analysis, where variables are usually evaluated, is an effective tool for determining the relationship between milk productivity and the feeding program [16]. These analyses evaluate which nutrients or feeding strategies are effective in increasing milk productivity, as well as the effects of other factors such as the genetic characteristics of cattle, environmental factors, and farm management on milk productivity, and make the necessary adjustments for optimum productivity [17]. Ensuring consistently high milk yield from dairy cattle depends on genetic characteristics, housing conditions, climate, and other environmental factors [18]. Statistical analyses and optimization methods have great potential to determine the most efficient production conditions by considering the different stages of the milk production process using a holistic approach [19,20]. Compared to traditional management approaches, such data-based methods make it possible to make scientific and systematic decisions in farm management [21].

Many studies in the literature examine the factors affecting productivity in dairy farming [22,23,24]. However, these studies generally focus on areas such as genetic selection, nutritional strategies, improvement of environmental factors, and management techniques, and most of these studies lack a combined consideration of the factors and their statistical optimization [5]. The basic hypothesis of this study is that milk yield is significantly affected by biological, environmental, and nutritional variables and that milk yield can be increased by optimizing these variables. The study aims to determine whether these variables affect milk yield positively or negatively.

While the breed, age, and yield of the animal have an essential effect on the milk performance of the animal in the success of dairy cattle farms, factors such as the capacity of the farm, the number of animals raised, the quality of the shelters, the experience and education level of the breeders, the qualified labor force used, and the amount and composition of concentrated feed in the ration determine the organizational quality of dairy cattle farms [25,26]. In dairy cattle, just as in the lives of all other living things, environmental conditions create a complex equation [27]. The components of this complex equation are mainly counted as feeding, sheltering, care, and climate factors [28]. Birth date, insemination date, calving date, lactation start date, dry-off date, first calving date, calving interval, lactation order, various farm factors, and climatic factors directly affect the milk yield of animals [29]. For effective breeding selection, the effects of environmental factors should be eliminated, and only the variation resulting from the genotypic effects of animals should be determined [30].

In many studies on milk productivity, data have been processed by considering seasonal effects, such as air temperature, relative humidity, and wind [31,32,33]. These factors are critical environmental variables that can affect the milk yield of animals [34]. High temperatures negatively affect milk productivity and can cause animals to experience heat stress, which can negatively affect milk production [35]. Similarly, one study emphasized that high relative humidity can also reduce the comfort level of animals and, therefore, milk yield [36]. In addition, the effects of wind on milk productivity should not be ignored [32]. Cold winds mainly affect the metabolism of animals and reduce milk yield [37].

This study aims to determine the most suitable conditions by considering biological, environmental, and nutritional factors affecting milk productivity, together with multivariate statistical optimization methods. In this respect, the study aims to increase milk yield, optimize dry matter consumption, and make resource use more efficient.

This article consists of six main sections. The introduction section of the study emphasizes that the factors affecting milk productivity are generally considered independently in the literature and that optimization methods are not used sufficiently. The second section explains that the variables affecting milk production are analyzed using linear regression and statistical optimization techniques, and the most suitable input variable values are determined. In the third section of the study, the results section, the statistical data obtained are presented, and the effects of factors such as TMR dry matter ratio, lactation period, gestation period, and air temperature on milk yield are detailed. The study’s findings are compared with the literature in the discussion section, and practical applications to increase milk production are discussed. Finally, in the conclusions section, it is stated that the optimization model contributes to increasing milk yield by determining the most suitable independent variable values, and suggestions for future research are presented.

## 2. Materials and Methods

**Figure 1:**
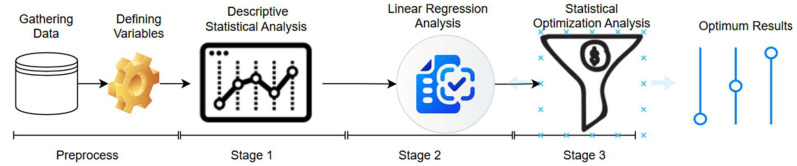
Stages of the study method.

This study aims to statistically analyze and optimize the biological, environmental, and nutritional factors that maximize milk productivity. In this context, the factors affecting milk productivity were examined using linear regression and other statistical optimization methods, and these factors’ optimum values were determined. The developed model will be used to make decisions to ensure higher efficiency in milk production processes. The method part of this study consists of three stages. The stages of the study method are shown in Figure 1. The preliminary stage of this study includes the process of collecting and sorting data. The first stage involves introducing datasets by obtaining descriptive statistical data on the factors that provide the data for the study. The second part of the method consists of calculating the factors’ statistical significance levels and magnitudes using linear regression analysis. Finally, creating an optimization model that will maximize milk productivity constitutes the third stage of the method part of this study.

The dataset used in our study belongs to a large-scale dairy farm located in western Türkiye. Environmental data, such as average temperature, humidity, and the temperature–humidity index (THI) in the region during the study period, were obtained from local meteorological stations and considered in the analysis. The barns on the farm are open systems with natural ventilation; fans and sprinklers are used for cooling in the summer months. The animals are kept in a free-range system and fed thrice daily. Rations are prepared using the total mixed ration (TMR) method and contain roughages, such as corn silage, alfalfa silage, and wheat straw, and concentrated feeds, such as corn, barley, sunflower meal, and factory feed. The roughage–concentrate ratio is adjusted according to the lactation period, with 55% roughage and 45% concentrate on average. Feeding management is optimized daily according to feed residue, and clean water is constantly provided to the animals.

Independent variables include total mixed ration (TMR), dry matter ratio, lactation number, lactation day number, pregnancy day number, cow age, and air temperature. These variables have numerical data types and are measured in percentages, numbers, and time units. In particular, the TMR dry matter ratio is expressed as a percentage, while lactation number and lactation and pregnancy day numbers are considered numerical values. The age variable represents the monthly age of the cows. Air temperature, an independent variable measured in degrees, was used to evaluate the effect of environmental factors (humidity, wind speed, etc.). On the other hand, dependent variables were determined as milk yield and dry matter (DM). Milk yield represents a numerical value measured in 24 h, while dry matter was calculated as unit/cow. In general, it shows that many factors that can affect the milk productivity of cows are being examined, and the relationships between these factors will be evaluated based on statistical analysis.

**Figure 2:**
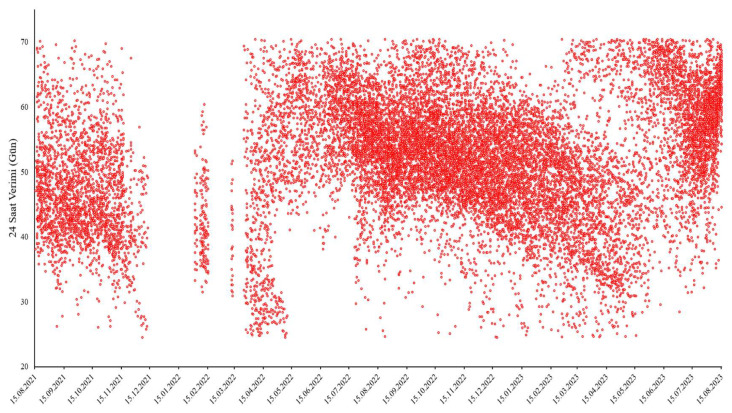
Daily milk yield during the period considered.

**Figure 3:**
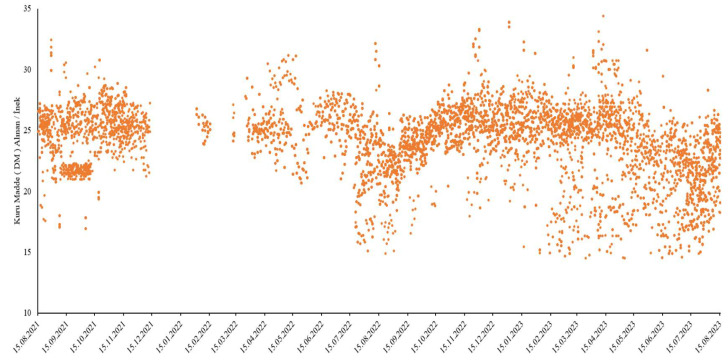
Dry matter (DM) intake/cow during the period considered.

Scatter plot images of the dependent variables, daily milk yield, and dry matter (DM) are shown in Figure 2 and Figure 3.

Figure 2 shows the distribution of daily milk yield during the period examined within the scope of the research. When the graph is reviewed, it is observed that milk yield is concentrated in a particular range, but there are also some extreme values. The average daily milk yield is determined to be 51.856 L, and when the data distribution is examined, it is seen that most observations are concentrated around this value. However, the extreme values determined as a minimum of 24.5 L and a maximum of 70.4 L indicate that some animals are more affected by environmental or biological factors. In addition, the slight negative skewness of the distribution shows that the milk yield of some cows is above average, but most animals remain at lower levels. This situation emphasizes the importance of optimization studies on milk yield and reveals the necessity of considering individual factors in farm management.

Figure 3 shows the distribution of the daily dry matter consumption of cows. The average dry matter consumption was calculated as 24.233 units, and it was observed that most observations were close to this value. However, the consumption values varying between a minimum of 14.51 and a maximum of 34.4 units indicate that individual differences play an important role. These differences may vary depending on the cows’ age, lactation stage, and metabolic requirements, as well as environmental factors. When the graph is examined, it is seen that the data have a slightly negative skew, and some cows have lower dry matter consumption than the average. In addition, the existence of individuals with high dry matter consumption reveals the importance of how energy needs are met with different feeding strategies. This analysis contributes to a better understanding of the relationship between milk yield and nutrient consumption and emphasizes the necessity of balanced feeding programs in farm management.

Using statistical optimization, this study analyzed the effects of various biological, environmental, and nutritional factors on the productivity of raw milk obtained from a dairy farm. All 50 animals analyzed in the study were Holstein breeds. The age range of the animals was between 3 and 6 years, and it was ensured that they had similar daily milk yield (DIM) during the lactation period in which they were included in the study. The average DIM value was 90 days, and the fact that all animals were between the first and fourth lactation ensured the homogeneity of the sample. The dataset was collected for two years, and the environmental conditions were similar throughout the study. The same management practices were maintained on the same farm, and there were no changes in feeding management and infrastructure conditions. Environmental data, such as air temperature, relative humidity, and wind speed, were obtained from local meteorological stations in both years, and temperature–humidity index (THI) calculations were made. In addition, the shelters are open systems, and fan and sprinkler systems are used for natural ventilation and cooling during the summer months. This study used a statistical optimization method to analyze the actual milk yield and the costs incurred for milk yield and to obtain optimum data with the optimization model.

Statistical optimization representation is an approach in which simplified methods are combined with distribution techniques to provide a process or system flow. The statistical calculation method is frequently applied in regular transitions by analyzing complex systems and optimizing these systems to the most efficient balance. This process is carried out by analyzing the variables in the system; appropriate models are created in line with the obtained data, processes are organized based on the system’s requirements, and the relevant parameters are adjusted most appropriately so that the targeted functions reach the optimum level. Statistical optimization is a critical approach offering a comprehensive and wide range of services, from industrial processes to production activities, sales and marketing methods, and other operational activities [38]. Finally, all statistical analyses were performed using MINITAB software 21.1.0. The relevant subheadings explain the statistical methods and analysis steps used in detail.

The second step of the study methodology is regression analysis, which aims to estimate the relationship between the dependent and independent variables [39]. In the regression analysis, the observation values of the dependent and independent variables are first analyzed, and a regression curve expressing this relationship is created. Then, parameter estimation is made based on certain assumptions, and hypothesis tests are performed on the model parameters. In linear regression analysis, a regression equation is established that expresses the relationship between the dependent variable and one or more independent variables. This equation is usually written in the following form [40]:

(1) $$y_{ijk}=\delta_{0}+\sum \delta_{j}x_{j}+\sum \sum \delta_{ij}x_{i}x_{j}+\epsilon_{ijk}$$

where $y_{ijk}$ is the dependent variable (e.g., milk productivity), $x_{j}$ and $x_{i}$ are independent variables, $\delta_{j}$ and $\delta_{ij}$ are expressed as regression coefficients, and $\delta_{0}$ represents the regression constant term. The coefficients show the effects of the independent variables on y. $\epsilon_{ijk}$ is the error term; it represents the random errors resulting from other unobservable model factors.

The optimization model is included in the last section of the study’s methodology. It is considered a valuable method for measuring the effects of these factors on milk productivity by using it to determine the factors affecting milk productivity. Optimization modeling is a mathematical method that guides the effective reaching of the determined target; this type of modeling is mainly used to increase or decrease the targets with limited data and certain constraints to the maximum level [41].

The objective function equation for the statistical optimization model is derived from the regression equation. Constraints are defined as the input parameters used in the regression analysis. In the optimization model, two different objective functions (maximum and minimum) are run for the same constraints. For this reason, this mathematical modeling, which has a two-way objective function, acts as a nonlinear optimization model [42]. The following formulas are used as examples of two-objective optimization models [43]:

(2) $$Min\;y=\delta_{0}+\sum \delta_{j}x_{j}+\sum \sum \delta_{ij}x_{i}x_{j}+\epsilon \;Max\;y=\delta_{0}+\sum \delta_{j}x_{j}+\sum \sum \delta_{ij}x_{i}x_{j}+\epsilon \;\;s.t.\;\;\;\;\;\;\;\;\;\;\;\;\;\;\;\;\;\;\;\;\;\;\;\;\;\;\;\;\;\;\;\;\;\;\;\;\;\;\;\;\;\;\;\;\;\;\;\;\;\;\;\;\;\;\;\;\;\;\;\;\;\;\;\;\;\;\;\;\;\;\;l_{b}\leq \;x_{j}\leq u_{b}\;x_{j}\geq 0$$

where the terms represent the lower and upper limits of constraints $l_{b}$ and $u_{b}$, and the symbol $x_{i}$ and $x_{j}$ represents the type of constraints.

## 3. Results

Determining and analyzing the factors affecting milk productivity is essential for the sustainability of milk production. Milk production is a complex process that is shaped by the interaction of biological, environmental, and nutritional factors. In particular, variables such as total mixed ration (TMR) dry matter ratio, lactation period, gestation period, cow age, and environmental factors are among the determining factors in milk production. This study analyzed biological and ecological variables to maximize milk yield and optimize dry matter consumption. The statistical and optimization data obtained in this section provide important clues for increasing efficiency in farm management.

### 3.1. Descriptive Statistics

**Figure 4:**
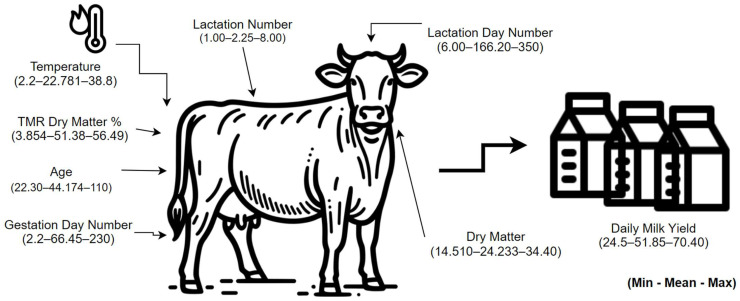
Minimum–mean–maximum values of the defined dependent and independent variables.

The data obtained for the dependent and independent variables were considered for a certain period. Seventeen thousand and five hundred data for each variable were analyzed. Understanding the distributions and basic statistical properties of the independent variables affecting milk yield and dry matter intake is of critical importance for the reliability of the analyses. Therefore, descriptive statistics were calculated for the independent and dependent variables used in the study, and the mean, standard deviation, variance, quartile values, and minimum and maximum values of the variables were determined. These analyses help us understand which factors show more variability in the milk production process and how these variables are related to milk yield. It was observed that factors such as lactation number, pregnancy period, and environmental temperature showed a wide distribution, and the effects of these variables on milk yield were examined in more detail (Figure 4).

The data collected during this period were analyzed using descriptive statistics, and some basic statistical results are shared in Table 1. These statistics reveal essential features of each variable, such as mean, standard error mean, standard deviation, variance, minimum, quartiles (Q1, median, Q3), maximum values, skewness, and kurtosis. First, the 24 h milk yield was determined to be 51.856 L. The standard deviation showed a significant variety with 8.99 L, while the variance was 80.979. The given milk yield’s lowest value was 24.5 L, and the highest value was 70.4 L. The lowest first quartile (Q1) among the quartiles was 46 L, while the median (Q2) was 52.3 L. The third quartile (Q3) represents the upper limits of milk yield, with 58.2 L. When these data are examined, it is seen that the distribution of milk yield is generally concentrated at medium and high levels, but there are also some exceptionally high values. The skewness value was calculated as −0.30, indicating a negative distribution.

The TMR dry matter (DM) ratio is another important variable that can directly affect milk yield, and it was determined to be 51.379% on average. The standard deviation around this value is 1.963, meaning that the TMR dry matter ratio generally varies between 50% and 56%. While the minimum value is 46.770%, the maximum value is 56.490%. With a Q1 value of 50.190%, a median of 50.790%, and a Q3 value of 51.740%, this variable’s distribution is relatively narrow, meaning that the dry matter ratio in most cows is concentrated in a particular range. The skewness value of 1.18 shows a positive trend; this indicates that there are mostly higher dry matter ratios and that this variable exhibits a right-skewed distribution. The kurtosis value is 0.54, indicating less intense extreme values around the mean of this variable.

For the dry matter intake/cow variable, the mean was calculated as 24.233 units, and the standard deviation was 2.776 units. This shows that the average dry matter intake per cow was around 24 units, but some cows may have higher or lower values. The minimum value was determined as 14.510 units, and the maximum as 34.4. The quartiles were 23.050, 24.750, and 25.870 units, respectively. This distribution shows that dry matter intake for most cows was in the range of 20–30 units. The skewness was determined as −0.75, indicating a negative trend and more low-value observations. The kurtosis value was 1.33, indicating a high density, meaning that most observations were close to the mean value.

The lactation number, one of the other independent variables, was calculated with a mean of 2.2520 and a standard deviation of 12.998. The lactation number indicates how long cows give milk, and these values are observed to spread over a wide range. The minimum value is 1, and the maximum value is 8. This wide distribution shows that the lactation number reflects the diversity of cows’ ages and production cycles. The average number of lactation days was calculated as 166.20, and the number of gestation days was 66.454. The standard deviations in these variables are 90.87 and 71.433, respectively. While the number of gestation days varies between 1 and 230, it is observed that the number of lactation days is also quite variable, and some cows have very long lactation periods. The average age was 44.174 months, and the average air temperature was 22.781 °C. The skewness and kurtosis values show that the distribution in these variables is quite balanced and that the extreme values are limited.

Descriptive statistics of other variables according to 5 frequency groups of each age group of the animals subject to the study are given in Table 2. In the evaluation made according to cow age groups, it is seen that the average milk yield (55.98 L/day) of animals between 62.4 and 82.3 months is at the highest level. The TMR dry matter ratio is also at the lowest level in this group (50.68%), which supports the positive effect of low TMR dry matter ratios on milk yield. However, it was observed that milk yield decreased significantly (45.96 L/day), and lactation number was the highest (7.20) in the oldest group, between 102.3 and 110 months. This shows that increasing lactation number with age decreases milk yield after a certain level. The number of lactation days also fluctuates with age, and the most extended period was observed in the 42.4–62.3 age group, with an average of 175.68 days. The pregnancy duration increases with age, reaching its highest value of 133.82 days in the 82.4–102.3 age range.

Depending on other variables, DM (dry matter) consumption is relatively stable among age groups; the highest consumption was observed in the 82.4–102.3-month group (24.65 units). The TMR dry matter ratio is also high in this age group (52.92%), suggesting that TMR content is directly related to dry matter consumption. When the temperature variable is examined, the average temperature is 23.06 °C in the younger age groups (22.3–42.3 months), while the temperature level decreases relatively in the older groups. This reveals no direct relationship between temperature and age group, but environmental effects may produce different results according to age group. The data show that age, lactation number, and TMR dry matter ratio, in particular, have a complex interaction with milk yield and dry matter consumption.

### 3.2. Regression Analysis

There are two different dependent variables in this study. For this reason, regression analysis was performed for these variables, and variance analysis results were calculated. The regression analysis for daily milk yield is given in Table 3.

Table 3 presents the ANOVA (analysis of variance) results on the daily milk yield variables. ANOVA tests the effect of independent variables (TMR dry matter ratio, lactation number, lactation days, gestation days, cow age, and temperature) on milk yield. F-value and *p*-value calculations were calculated for each independent variable. Here, it is seen that all independent variables are statistically significant because the *p*-values are less than 0.05 for each, indicating that each variable has a significant effect on the daily milk yield. Contents such as TMR dry matter ratio (%), gestation days, and lactation days have very high F-values, indicating that these variables strongly affect milk yield. The F-value of the TMR dry matter ratio is relatively high, at 978.26, and its *p*-value is 0.001. This indicates that this variable has a powerful effect on milk yield.

**Figure 5:**
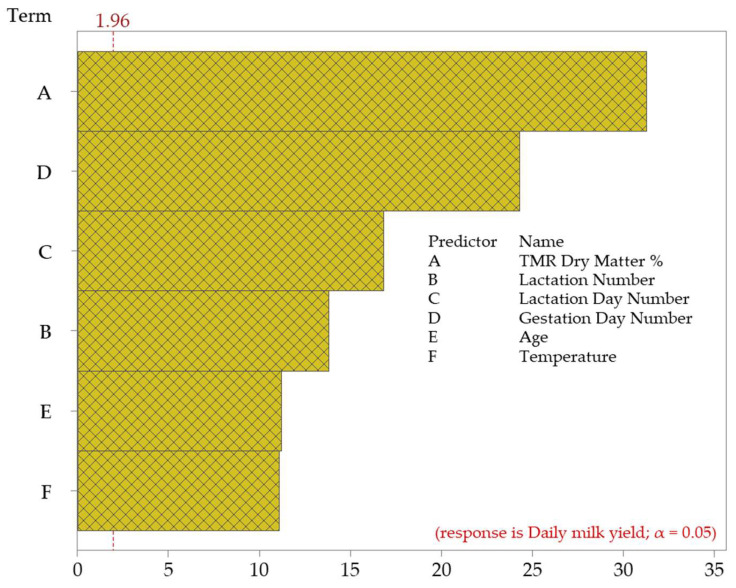
Pareto chart of standardized effects on daily milk yield.

Similarly, lactation number, gestation number, cow age, and temperature significantly affected daily milk yield. The effect of lactation number reached a high value with an F-value of 190.63, and the impact of gestation number reached a high value of 591.09. The *p*-values of these variables ranged between 0.001 and 0.002, indicating that each significantly affected the daily milk yield. In addition, it was observed that the temperature variable also affected milk yield, but this effect was not as strong as the other variables (F-value 123.15). These findings reveal that milk yield is related to environmental and biological factors and that the effects of these factors on production should be evaluated carefully. The Pareto chart showing the impact of the independent factors on the dependent variable is given in Figure 5.

The regression analysis of dry matter, another dependent variable, to measure the effect of the independent variables is given in Table 4. It was designed to examine whether each independent variable affecting the amount of dry matter is statistically significant. All independent variables were observed to affect the dry matter amount in total, but some variables showed this effect more strongly. First, the general regression analysis results show that a model explains 100% of the total variance, and the *p*-value is 0.001. This reveals that the model is generally significant and that the independent variables affect dry matter. The F-values and *p*-values for each independent variable are important data showing how effective certain variables are on dry matter.

The TMR dry matter ratio (%) strongly affects dry matter amount, indicating a high value with an F-value of 256.97. In addition, the *p*-value was calculated as 0.001, confirming that this variable significantly affects the amount of dry matter. The number of lactations is a variable with less effect on dry matter because the F-value was only 2.10, and the *p*-value was not statistically significant at 0.148. This shows that the number of lactations does not significantly affect the amount of dry matter. The number of lactation days has a powerful effect, reaching a very high value of 1523.69 and a *p*-value of 0.001. This shows that as the lactation period increases, the amount of dry matter also increases, and this variable plays an important role.

**Figure 6:**
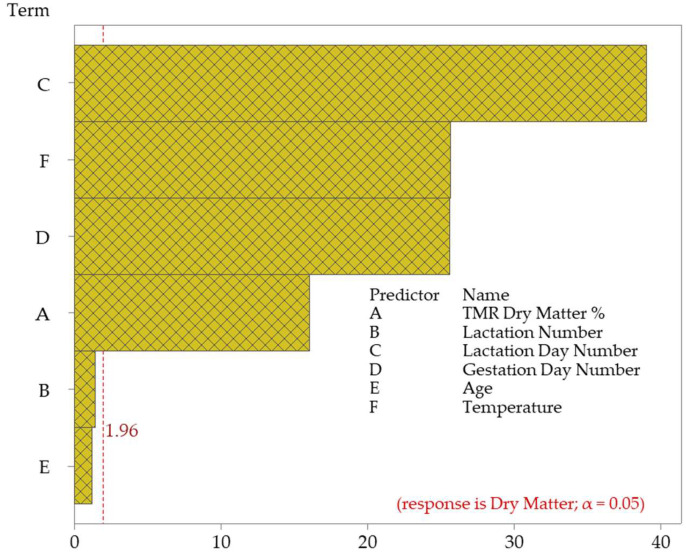
Pareto chart of the standardized effects of dry matter.

The number of pregnancy days and air temperature also significantly affect dry matter. The F-value of the number of pregnancy days was 655.94, and the air temperature was 659.34, both showing a strong effect. The *p*-values of each of these variables were calculated as 0.001, meaning that both factors significantly affect dry matter. On the other hand, the effect of the cows’ age on dry matter remains weaker. The F-value of the age variable is 1.51, and the *p*-value is 0.220, which indicates that age does not significantly affect the amount of dry matter. In general, it is possible to say that factors such as the TMR dry matter ratio, number of lactation days, number of pregnancy days, and air temperature are the most important variables affecting the amount of dry matter. The Pareto chart showing the effect of the independent factors on the dependent variable is given in Figure 6.

The regression equations for both dependent variables, which include independent variables with different coefficients, are given below as Equations (3) and (4).

(3) DailyMilkyield=109.87−0.9148 TMR Dry Matter %+3.753 Lactation Number                                  −0.02229 Lactation Day Number−0.03834 Gestation Day Number−0.2440 Age−0.10651 Temperature                                        

(4) Dry Matter=16.202+0.15626TMR Dry Matter %+0.1311 Lactation Number                        +0.017244 Lactation Day Number−0.013458 Gestation Day Number              −0.00891 Age+0.08213 Temperature                     

Equation (3) explains how daily milk yield (24 h yield) is affected by various factors. According to this equation, each 1% increase in the TMR dry matter ratio leads to a decrease in daily milk yield of 0.9148 L. This negative relationship shows that a high TMR dry matter ratio can negatively affect milk yield. The number of lactations positively affects milk yield; each lactation number increases the daily milk yield by 3.753 L. The number of lactation days appears to have a negative effect on daily milk yield, as each increase in lactation day leads to a decrease in milk yield by 0.02229 L. Factors such as the number of days of gestation, age, and air temperature also negatively affect the daily milk yield. Each increase in the number of days of gestation leads to a decrease in milk yield by 0.03834 L, while cow age leads to a reduction of 0.2440 L. In addition, each degree increase in air temperature is associated with a decrease in milk yield of 0.10651 L. This regression equation shows that milk yield is greatly affected by environmental and biological factors and that yield increases significantly as the number of lactations increases. Still, other factors, especially the dry matter ratio and environmental conditions (temperature), can negatively affect yield.

Equation (4) examines which factors affect the dry matter intake per cow. Each 1% increase in the TMR dry matter ratio increases the amount of dry matter intake per cow by 0.15626 units. This positive relationship shows that cows consume more dry matter as the dry matter ratio rises. Each increase in the number of lactations increases the amount of dry matter intake by 0.1311 units, which indicates that cows consume more dry matter during the lactation period. The number of lactation days also increases dry matter intake, but its effect is less (0.017244 units increase). On the other hand, the number of days of gestation, age, and air temperature decreases the effects of dry matter intake. Each increase in the number of days of gestation decreases dry matter intake by 0.013458 units and age by 0.00891 units. Air temperature decreases dry matter intake by 0.08213 units, indicating that high temperatures reduce the dry matter consumption of cows. In general, these equations show that the dry matter intake of cows is shaped by various factors, especially environmental conditions such as dry matter rate, lactation number, and temperature, which have significant effects.

### 3.3. Statistical Optimization Analysis

Equations (1) and (2) constitute the objective functions of the statistical optimization model. Both objective functions have the same constraints and limits depending on the criteria. The equation obtained with the is a nonlinear optimization model. The optimization model created for this study is given in Equation (5):

(5) $$Max\;Eq\;(1)-Daily\;Milk\;Yield\;Min\;Eq\;\left(2\right)-Dry\;Matter\;\;s.t.\;\;\;\;\;\;\;\;\;\;\;\;\;\;\;\;\;\;\;\;\;\;\;\;\;\;\;\;\;\;\;\;\;\;\;\;\;\;\;\;\;\;\;\;\;\;\;\;\;\;\;\;\;\;\;\;\;\;\;\;\;\;\;\;\;\;\;\;\;\;\;\;\;\;\;\;\;\;\;17.51\leq TMR\;Dry\;Matter\;\%\leq 56.49\;1689\leq Lactation\;Number\leq 8\;6\leq Lactation\;Day\;Number\leq 350\;1\leq Gestation\;Day\;Number\leq 230\;22.30\leq Age\leq 110\;\;\;\;\;\;\;\;\;\;2.2\leq Temperature\leq 38.8$$

Equations (3) and (4) represent the objective functions of the statistical optimization model used in this study. Equation (3) aims to maximize the daily milk yield, while Equation (4) seeks to minimize the dry matter intake of cows. These two equations are solved under the same constraints and limitations, while each tries to achieve a different goal. Maximizing daily milk yield aims to increase milk production while minimizing dry matter intake, which ensures that cows are fed more efficiently and resources are used more effectively. Such a model is crucial for developing optimal nutrition and productivity strategies in farm management. Optimizing two different goals together is of great value, especially in terms of balancing milk yield and nutrition costs.

The equation obtained emerges as a nonlinear optimization model. This shows that the model has a structure with complex and nonlinear interactions between variables rather than simple linear relationships. Such models provide more appropriate solutions for situations where many variables and interactions are considered, which are commonly encountered in real-world problems. The optimization model helps establish a more efficient and sustainable production system by considering two goals: increasing milk yield and reducing dry matter intake. Thus, farmers can obtain higher yields from milk yield and use nutritional resources more effectively. This approach offers an essential strategy for efficient food production and resource management.

Finally, the optimization model created with Equation (5) is solved under certain limitations (constraints). These constraints require each independent variable (such as the TMR dry matter ratio, lactation number, lactation days, pregnancy days, age, and temperature) to be within a specific range. The TMR dry matter ratio was between 46.77% and 56.49%, and the lactation number was between 1 and 8. In addition, physical limits and environmental conditions were considered, such as the age of the cows being between 22.30 months and 110 months and the temperature being between 2.2 °C and 38.8 °C. These constraints ensure that the model is realistic and applicable. This optimization model aims for the best results while considering physical and biological limitations, creating a solid foundation for real-world applicability. Such constraints make it possible to develop an optimized and sustainable solution for farm management. The optimum data of constraints and objective functions for the optimization model are shared in Table 5 and Table 6.

Table 5 shows the optimum values of the independent variables (constraints) obtained according to a specific optimization model. These values indicate that each factor is adjusted at the most appropriate level to provide maximum efficiency and sustainability. First, the TMR dry matter ratio is determined to be 46.77%. This refers to the increase in the dry matter ratio used in the cows’ nutrition to the optimum level. The dry matter ratio is a factor that directly affects nutritional efficiency; this ratio is adjusted to this level to provide an ideal balance for the cows’ health and milk yield. This optimum ratio aims to keep milk production at the highest level by considering feeding costs and resource efficiency.

Another constraint is the number of lactations; the optimum value is 5. This value means that the number of lactations in which the cows are most productive is 5. The number of lactations is an essential factor determining cows’ effect on milk production. A higher number of lactations allows the cows to produce milk for a more extended period. Still, the improvements and maintenance made after each lactation are also significant. Calculating the lactation number as five indicates that this number is ideal for reaching the optimum production level. Increasing the lactation number to this value indicates that the cows are adjusted to provide maximum efficiency.

Other independent variables included in the optimum values are the number of lactation days, the number of gestation days, the age of the cow, and the air temperature. The number of lactation days was determined to be 6, which indicates the time required for each lactation process to be carried out efficiently. The number of gestation days was selected as 230 days; this value is compatible with the standard gestation period of the cows and is a period that will not have a negative effect on milk production and health. The optimum value of cow age was calculated as 55.8 months, indicating the period when the cows are productive and most productive. Finally, the air temperature was determined to be 22 °C, suggesting the environmental temperature limit at which the cows perform best. Each constraint was carefully selected to maximize efficiency in farm management, and these optimum values are critical to ensuring sustainability in milk production.

Table 6 presents the statistical results of the optimum data obtained for the dependent variables (objective functions). The optimum milk yield was determined to be 61.145 L; this value’s margin of error (standard error) was 1.33. This is an essential measure of the reliability of the milk yield estimate. In addition, the milk yield’s 95% confidence interval (CI) is between 60.79 and 73.01 L. This interval represents the highest and lowest estimated values, compatible with the actual value of the optimum milk yield obtained. The 95% prediction interval (PI) is between 55.82 and 84.98 L, indicating a 95% probability that a future observation will be within this interval. This wide prediction interval implies more uncertainty in the future values of milk yield, meaning that external factors can affect milk yield more.

The optimum dry matter intake was determined as 19.033 units; this value’s standard error value is 0.443. This also expresses the reliability of the estimates of dry matter intake. The 95% confidence interval (CI) of dry matter intake is between 17.014 and 19.752 units, which indicates how much the actual value of dry matter intake could be. This confidence interval shows that the model estimates dry matter intake quite reliably. In addition, the 95% prediction interval (PI) is between 13.026 and 22.741 units, which indicates the probability that future observations will be within this interval. This wider prediction interval suggests that there is more uncertainty in dry matter intake due to the effect of some variables and that individual cows may have different levels of dry matter intake.

Table 6 provides essential information showing the accuracy and reliability of the statistical calculations made for daily milk yield and dry matter intake. These statistical measurements help understand how robust and consistent the model is. Since optimizing the relationship between milk yield and dry matter intake is an essential goal in terms of productivity and resource management, such analyses allow for more informed and effective decisions to be made in farm management. At the same time, the wide range of prediction intervals will enable farmers to act in a way that considers uncertainty in developing strategies for future milk production and nutrition planning.

As a result, the optimum data presented in Table 5 and Table 6 allow for meaningful inferences regarding farm management and livestock productivity. The optimum values of the independent variables in Table 5 form the basis of a strategy aimed at increasing milk yield, while also demonstrating an approach aimed at optimizing dry matter intake. In particular, the TMR dry matter ratio was determined to be 46.77%, which is an ideal level for increasing the nutritional efficiency of cows. Increasing the lactation number to 8 indicates when cows achieve maximum productivity. At the same time, other constraints (age, temperature, number of days of gestation) were adjusted to the optimum level considering environmental and biological factors. These data show that the conditions required for sustainable and efficient milk production are provided and that farm management can achieve higher yields with these levels.

**Figure 7:**
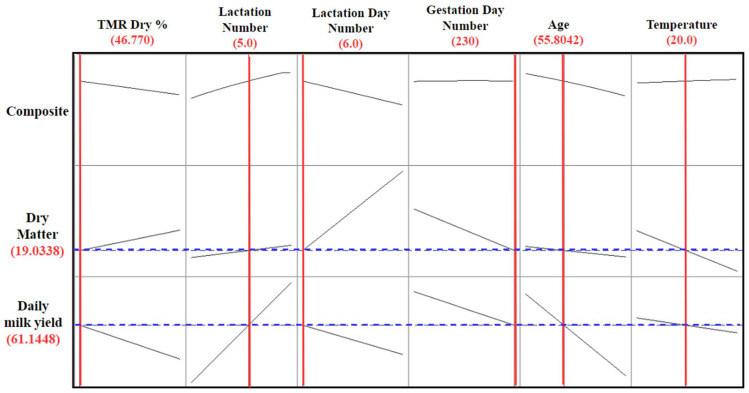
Optimum values of objective functions and constraints of the optimization model.

The statistical analysis results of the dependent variables in Table 6 confirm the reliability and effectiveness of this optimization model. The 24 h milk yield was calculated to be 61.1448 L, with a confidence interval between 60.79 and 73.01 L. At the same time, the dry matter intake was determined to be 19.033 units and offered a confidence interval between 19.014 and 18.752 units. These results show that both objective functions can be optimized with high accuracy based on the model’s estimates. However, the width of the estimated interval for milk yield contains a particular uncertainty about future productivity due to environmental factors. This situation emphasizes that farmers should consider possible variability in future production plans. In general, both tables provide data that will contribute to the establishment of an efficient production system by optimizing the balance between milk yield and dry matter intake. A visual representation of the optimization model that considers both objective functions and constraints is given in Figure 7.

Figure 7 visually presents the optimum values of the objective functions and constraints of the optimization model. The independent variables determined during the optimization process included the TMR dry matter ratio, lactation number, lactation day number, pregnancy day number, cow age, and air temperature. These variables were analyzed to balance their effects on dependent variables, such as milk yield and dry matter consumption. The model mainly aims to maximize milk yield while keeping dry matter consumption within certain limits. The optimization graph shows the ideal conditions for farm management by determining the optimum points of different independent variables. While the optimum TMR dry matter ratio for milk yield was determined to be 46.77%, the most efficient value for the lactation number was calculated as 5. These points are the recommended values to obtain maximum milk production while preserving the health and productivity of cows.

In this study, the limits of the independent variables were determined during the optimization process, and the best levels compatible with biological factors were calculated. Considering the effects of air temperature on milk yield, the optimum temperature value was determined to be 20.0 °C. This shows that appropriate environmental conditions should be provided to minimize heat stress. Similarly, when the number of lactation days is adjusted to the optimum level, milk yield and animal health are protected by balancing the metabolic load of cows. All these optimum data provide practical information to farmers and researchers, helping them make data-driven animal nutrition and management decisions. In this context, the balance between milk yield and feed consumption is best established thanks to the optimization model, providing a strong basis for sustainable and efficient production strategies in farm management.

## 4. Discussion

This study provides a comprehensive analysis of the factors affecting milk yield and dry matter intake. The findings revealed that various independent variables significantly affected milk yield and dry matter intake. The discussion section discusses the compatibility of these findings with the literature, their practical applications, and their potential implications for milk production. According to the data obtained, milk yield was calculated as an average of 51.856 L, with a minimum of 24.5 L and a maximum of 70.4 L. This wide range demonstrates the variable effects of different factors on milk yield.

This study determined that the average TMR dry matter ratio was 51.379%, with values ranging from 46.770% to 56.490%. According to the regression analysis results, each 1% increase in the TMR dry matter ratio causes a decrease of 0.9148 L in the daily milk yield. This study calculated the average lactation number as 2.252, and it varied from 1 to 8. According to the regression analysis, each increase in the number of lactations increases the daily milk yield by 3.753 L. However, productivity is thought to decrease in the later lactation periods, and the lactation number that reaches the optimum level is 8. This situation shows that milk production increases up to a certain point but decreases after a certain point due to the physiological limits of the cows. The study determined that the average number of lactation days was 166.20 days. According to the regression analysis, each increase in lactation days leads to a decrease of 0.02229 L in milk yield.

In the study, the number of days of pregnancy was determined to be 66,455 days, and it was seen that it varied from a minimum of 1 day to a maximum of 230 days. According to the regression analysis, each increase in the number of days of pregnancy leads to a decrease in milk yield of 0.03834 L. It was found that increasing air temperature reduces milk yield. In the study, the average air temperature was calculated as 22.781 °C and was determined to vary from a minimum of 2.2 °C to a maximum of 38.8 °C. According to the regression analysis, each degree increase in air temperature causes a decrease in milk yield of 0.10651 L.

The study determined that the average dry matter intake was 24.233 units, which varied from a minimum of 14.510 to a maximum of 34.400 units. According to the regression analysis, each 1% DM dry matter ratio increased the TMR dry matter intake by 0.15626 units. It was determined that an increase in lactation days increased dry matter consumption. According to the regression analysis, each increase in lactation days caused an increase of 0.017244 units in dry matter intake. The increase in the number of pregnancy days decreased the dry matter intake. In this study, it was determined that the number of days of pregnancy varied from 1 to 230 days. According to the regression analysis, each increase in the number of days of pregnancy decreased the dry matter intake by 0.013458 units.

The optimization model determined the most suitable levels of certain variables for maximum milk yield and optimum dry matter intake. The optimum independent variable values defined in the study are as follows: TMR dry matter ratio 46.77%, lactation number 5, lactation number 6, pregnancy number 230, age 55.8 months, and air temperature 20.0°C. The optimum values of the dependent variables were calculated as 61.145 L for milk yield and 19.033 units for DM dry matter intake.

This study has several limitations. First, the data for this study were collected from a single farm, which limits the generalizability of the findings. Differences in climate, farm management practices, housing systems, and herd genetics on other farms are likely to affect the applicability of the results. Future studies that include multiple farms under various environmental and management conditions are needed to confirm and extend these findings. Second, while the statistical optimization approach provides valuable insights, it assumes linear relationships among variables and simplifies complex biological interactions. Finally, the dataset included only Holstein cows with relatively homogeneous traits, and data from other breeds or mixed herds were not considered.

## 5. Conclusions

This study evaluated the effects of biological, nutritional, and environmental factors on milk yield using regression analysis and optimization modeling. The results revealed that the TMR dry matter ratio, lactation number and duration, pregnancy days, and ambient temperature significantly influenced milk production. While higher TMR dry matter ratios increased feed intake, they negatively affected milk yield, emphasizing the importance of balanced feeding strategies.

This study investigated the impact of various factors on milk yield and dry matter intake in dairy cows and developed an optimization model to enhance production efficiency. The average TMR dry matter ratio was 51.38%, and each 1% increase resulted in a 0.91 L decrease in milk yield, while increasing dry matter intake by 0.16 units. Although higher TMR dry matter ratios promoted feed consumption, they adversely affected milk production, highlighting the need for optimized feeding strategies. The lactation number averaged 2.25 and positively influenced milk yield by 3.75 L per additional lactation. However, excessive lactation numbers eventually reduced productivity. The average lactation period was 166.2 days, and each additional day reduced milk yield by 0.022 L while increasing dry matter intake by 0.017 units. Pregnancy duration showed a similar trend, where each added day decreased milk yield by 0.038 L. The ambient temperature ranged from 2.2 °C to 38.8 °C, with an average of 22.78 °C. Each 1 °C increase caused a 0.11 L reduction in daily milk yield, underlining the importance of mitigating heat stress.

Based on the optimization model, the ideal conditions for maximizing milk yield (61.15 L/day) and maintaining efficient dry matter intake (19.03 units/day) were as follows: 46.77% TMR dry matter ratio, five lactations, six lactation days, 230 days of pregnancy, 55.8 months of age, and 20 °C ambient temperature. These findings provide a quantitative basis for improving dairy farm management through balanced nutrition and environmental control. Environmental factors such as temperature also played a critical role, with rising temperatures associated with reduced milk yield, likely due to heat stress. The developed optimization model identified ideal conditions for maximizing milk yield and managing dry matter intake, offering a practical framework for enhancing dairy farm productivity through data-driven management. This study provides valuable insights into milk yield, although its results are based on data from only one farm. Therefore, caution should be exercised when generalizing the results to other farms with different management practices or environmental conditions.

## Figures and Tables

**Table 1:** Descriptive statistics of variables.

Variable	Milk Yield	TMR Dry Matter %	Dry Matter (DM)	Lactation Number	Lactation Day Number	Gestation Day Number	Age	Temperature
Mean	51.856	51.379	24.233	2.25200	166.200	66.4540	44.174	22.781
SE Mean	0.0680	0.0148	0.0210	0.00983	0.68700	0.54000	0.1220	0.0588
StDev	8.9990	1.9630	2.7760	12.9980	90.8700	71.4330	16.139	7.7750
Variance	80.979	3.8540	7.7050	16.8940	8258.18	5.10200	260.45	60.456
Minimum	24.500	46.770	14.510	1.00000	6.00000	1.00000	22.300	2.2000
Q1	46.000	50.190	23.050	10.0000	92.0000	1.00000	31.100	17.200
Median	52.300	50.790	24.750	20.0000	162.000	47.0000	41.000	22.200
Q3	58.200	51.740	25.870	30.0000	241.000	124.000	52.600	30.000
Maximum	70.400	56.490	34.400	8.00000	350.000	230.000	110.00	38.800
Skewness	−0.3000	1.1800	−0.7500	1.40000	0.10000	0.64000	1.2500	−0.1500
Kurtosis	−0.1900	0.5400	1.3300	2.38000	−1.04000	−0.98000	1.9500	−0.9200

**Table 2:** Descriptive statistics of other variables depending on cow age groups.

Variable	Age Frequency	Mean	StDev	Minimum	Maximum	Skewness	Kurtosis
Milk Yield	[22.3–42.3)	52.051	7.108	35.786	65.318	−0.269	0.013
	[42.4–62.3)	51.240	8.990	34.572	66.107	−0.191	−0.619
	[62.4–82.3)	55.982	5.060	48.371	62.450	−0.285	−0.375
	[82.4–102.3)	52.289	2.679	49.705	54.710	−0.052	−0.013
	[102.3–110]	45.963	1.855	44.332	47.743	0.152	−0.895
TMR Dry Matter (%)	[22.3–42.3)	51.794	1.873	49.202	55.933	0.974	0.813
	[42.4–62.3)	51.156	1.673	48.983	55.072	1.136	1.443
	[62.4–82.3)	50.679	0.825	49.740	52.120	0.393	0.778
	[82.4–102.3)	52.916	0.503	52.442	53.326	−0.050	−2.086
	[102.3–110]	50.346	0.292	50.086	50.613	−0.069	0.765
Dry Matter (DM)	[22.3–42.3)	24.229	2.446	17.795	29.593	−0.477	1.664
	[42.4–62.3)	23.973	2.871	17.627	29.351	−0.467	1.318
	[62.4–82.3)	24.293	1.935	21.406	27.171	−0.057	0.805
	[82.4–102.3)	24.648	1.231	23.505	25.781	0.007	0.901
	[102.3–110]	24.398	1.586	22.818	25.627	−0.294	0.735
Lactation Number	[22.3–42.3)	3.269	0.300	2.885	3.513	−0.845	4.522
	[42.4–62.3)	2.829	0.223	2.495	3.030	−0.819	2.316
	[62.4–82.3)	4.455	0.179	4.148	4.571	−1.311	0.932
	[82.4–102.3)	6.400	0.000	6.400	6.400	*	*
	[102.3–110]	7.196	0.000	7.196	7.196	*	*
Lactation Day Number	[22.3–42.3)	147.480	56.498	36.940	237.637	−0.533	1.312
	[42.4–62.3)	175.678	73.715	61.635	282.650	−0.247	−0.636
	[62.4–82.3)	4.455	0.179	4.148	4.571	−1.311	0.932
	[82.4–102.3)	133.819	2.265	131.600	135.739	−0.050	−2.269
	[102.3–110]	7.196	0.000	7.196	7.196	*	*
Gestation Day Number	[22.3–42.3)	50.569	46.393	1.139	138.851	0.718	0.331
	[42.4–62.3)	85.119	52.434	24.290	167.430	0.296	−0.791
	[62.4–82.3)	4.455	0.179	4.148	4.571	−1.311	0.932
	[82.4–102.3)	133.819	2.265	131.600	135.739	−0.050	−2.269
	[102.3–110]	7.196	0.000	7.196	7.196	*	*
Temperature	[22.3–42.3)	23.062	5.696	11.053	33.204	−0.224	−0.139
	[42.4–62.3)	21.444	6.238	10.029	31.432	−0.129	−0.594
	[62.4–82.3)	4.455	0.179	4.148	4.571	−1.311	0.932
	[82.4–102.3)	133.819	2.265	131.600	135.739	−0.050	−2.269
	[102.3–110]	7.196	0.000	7.196	7.196	*	*

* Not available.

**Table 3:** ANOVA data for daily milk yield.

Variable	DF	Adj SS	Adj MS	F-Value	*p*-Value
TMR Dry Matter (DM) %	1	523,620	52,362.3	978.26	0.001
Lactation Number	1	10,204.0	10,203.6	190.63	0.001
Lactation Day Number	1	15,140.0	15,139.9	282.85	0.002
Gestation Day Number	1	31,638.0	31,638.3	591.09	0.001
Age	1	6717.00	6716.60	125.48	0.001
Temperature	1	6592.00	6591.80	123.15	0.002

**Table 4:** ANOVA data for dry matter.

Variable	DF	Adj SS	Adj MS	F-Value	*p*-Value
TMR Dry Matter (DM) %	1	1528.0	1527.69	256.970	0.001
Lactation Number	1	12.000	12.4600	2.10000	0.148
Lactation Day Number	1	9058.0	9058.19	1523.69	0.001
Gestation Day Number	1	3899.0	3899.49	655.940	0.001
Age	1	9.0000	8.95000	1.51000	0.220
Temperature	1	3920.0	3919.72	659.340	0.001

**Table 5:** Optimum data for independent variables (constraints).

Independent Variables (Constraints)	Optimum Data
TMR Dry Matter (DM) %	46.77
Lactation Number	5.000
Lactation Day Number	6.000
Gestation Day Number	230.0
Age	55.80
Temperature	22.00

**Table 6:** Statistical-based optimum data for dependent variables (objective functions).

Response	Fit	SE Fit	95% CI	95% PI
Milk Yield	61.145	1.33	(60.79; 73.01)	(55.82; 84.98)
Dry Matter (DM)	19.033	0.443	(19.014; 18.752)	(13.026; 22.741)

## Data Availability

The data presented in this study are available on request from the corresponding author.
